# Anti-biofilm Activity as a Health Issue

**DOI:** 10.3389/fmicb.2016.00592

**Published:** 2016-04-26

**Authors:** Sylvie Miquel, Rosyne Lagrafeuille, Bertrand Souweine, Christiane Forestier

**Affiliations:** ^1^Laboratoire Microorganismes : Génome et Environnement – UMR, CNRS 6023, Université Clermont AuvergneClermont-Ferrand, France; ^2^Service de Réanimation Médicale Polyvalente, CHU de Clermont-Ferrand, Clermont-FerrandFrance

**Keywords:** anti-biofilm, biofilm, probiotics, pathogens, lock solution

## Abstract

The formation and persistence of surface-attached microbial communities, known as biofilms, are responsible for 75% of human microbial infections (National Institutes of Health). Biofilm lifestyle confers several advantages to the pathogens, notably during the colonization process of medical devices and/or patients’ organs. In addition, sessile bacteria have a high tolerance to exogenous stress including anti-infectious agents. Biofilms are highly competitive communities and some microorganisms exhibit anti-biofilm capacities such as bacterial growth inhibition, exclusion or competition, which enable them to acquire advantages and become dominant. The deciphering and control of anti-biofilm properties represent future challenges in human infection control. The aim of this review is to compare and discuss the mechanisms of natural bacterial anti-biofilm strategies/mechanisms recently identified in pathogenic, commensal and probiotic bacteria and the main synthetic strategies used in clinical practice, particularly for catheter-related infections.

## Introduction

Biofilms are multimicrobial communities enclosed in self-synthetized polymeric matrices, attached to biotic or abiotic surfaces. Eighty percent of the world’s microbial biomass are found in the biofilm state, and sessile cells are thus considered as the predominant mode of life for microorganisms in nature. These cells frequently express phenotypes different from their non-adherent planktonic counterparts, with a high capacity to colonize new surfaces and a high tolerance to exogenous stress (Donlan and Costerton, 2002; Macfarlane and Dillon, 2007). Depending on the microbial species and their localization (environmental/biomedical/industrial), biofilms can be either beneficial or detrimental for humans. According to the National Institutes of Health, more than 75% of microbial infections that occur in the human body are promoted by the formation and persistence of biofilms. Some bacterial biofilms, such as the intestinal microbiota, also play protective and functional roles. Intestinal commensal and beneficial bacteria–bacteria interactions are directly involved in host homeostasis (Wrzosek et al., 2013). In human health, an imbalance of microbiota, called dysbiosis, is associated with several diseases (Martín et al., 2014a). This correlation is in part due to bacterial interplay between members of bacterial communities such as group effect, cooperation, kin competition, genetic expression profiles, and phenotypic diversification (Rendueles and Ghigo, 2015) that could be encompassed by the adjective “anti-biofilm”. Interference interactions have already inspired the design of alternatives to antibiotics in the fight against pathogenic microorganisms (Rasko and Sperandio, 2010). Recently, major challenges and opportunities in this field were addressed during the workshop “Biofilms, Medical Devices, and Anti-Biofilm Technology” (Phillips et al., 2015). Many medical device-associated and persistent infections can be attributed to biofilm-associated microbes. To tackle the overarching public health issue of the contribution of biofilms to health care-associated infections it was suggested that clinicians and health care workers should be more closely involved in their detection and treatment. It was also suggested that the applied science of biofilm formation and prevention would provide greater knowledge of the contamination of medical devices. Some answers are to be found in the development of the anti-biofilm activities of beneficial microbes and/or the understanding and diversion of the anti-biofilm capacities of pathogenic bacteria. In this review, after establishing a definition of the term anti-biofilm, we will focus on bacterial anti-biofilm activities with examples of probiotic and pathogenic bacteria. With reference to clinical examples, we will then discuss the use, challenges and limitations of anti-biofilm strategies.

## Anti-Biofilm Activity: What Does it Mean?

**FIGURE 1:**
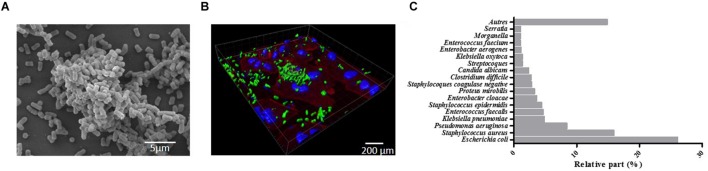
**Four-hour-old biofilm of *Klebsiella pneumoniae*. (A)** scanning electron microscopy (SEM) observation of the biofilm formed on Thermanox slide performed as previously described (Goncalves et al., 2014). **(B)** Confocal microscopic observations of the biofilm (green) formed onto A549 pulmonary cell monolayer stained with Phalloidin (red) and DAPI (blue). Observations were performed as previously described (Marquès et al., 2015). **(C)** Relative percentage of the most frequent micro-organisms associated with nosocomial infections in France (adapted from RAISIN, 2015).

Biofilms were initially defined as structured communities of bacterial cells enclosed in self-produced polymeric matrices and adherent to inert or living surfaces (Costerton et al., 1999). Later, it became obvious that biofilms exhibit altered phenotypes compared with corresponding planktonic cells, especially with regard to gene transcription (Lindsay and von Holy, 2006). Biofilms are ubiquitous and nearly all species of microorganisms, bacteria, fungi, yeasts, algae, protozoa, and viruses are able to adhere to surfaces and/or to each other to form biofilms (Wingender and Flemming, 2011). Biofilms formed by pathogenic bacteria are the most extensively documented, such as *Klebsiella pneumoniae* biofilms seen in **Figure 1** on abiotic (1A) and biotic surfaces (epithelial cell monolayer; 1B). Biofilms are increasingly recognized by the public health community as an important source of pathogens (Donlan and Costerton, 2002; Wingender and Flemming, 2011). They are involved in specific infectious diseases such as osteomyelitis, otitis media, peridontitis, and dental caries (Costerton et al., 1999) and in chronic diseases such as pulmonary infections of cystic fibrosis patients. They are also involved in nosocomial infections due to opportunistic pathogens, especially urinary tract, lower respiratory tract, and surgical site infections and bacteremia, and mostly when invasive medical device are being used. In 2012, a prevalence survey of 1,938 healthcare facilities and 300,330 patients carried out by the French association RAISIN “Réseau d’alerte, d’investigation et de surveillance des infections nosocomiales” showed that the most frequent microorganisms associated with nosocomial infections (RAISIN, 2015) were *Escherichia coli*, *Staphylococcus aureus* (38.1% resistant to methicillin, MRSA), *Pseudomonas aeruginosa*, and *K. pneumoniae*, all of which are high biofilm producers (**Figure 1C**).

Biofilms pose significant clinical problems because sessile bacterial cells are inherently recalcitrant to antimicrobial agents such as antibiotics (for review, Lebeaux et al., 2014). Several factors are responsible for the biofilm-associated resistance, including the density and the physiological state of the cells, but also the physical structure of the biofilm. Exopolysaccharides and extracellular DNA (eDNA) of the biofilm matrix can act as a barrier to diffusion and thus reduce penetration of antibiotics into biofilms. The effectiveness of this barrier varies between antibiotics; tetracyclines, rifamycins, fluoroquinolones, and daptomycin penetrate better than beta-lactams, aminoglycosides, and glycopeptides (Stewart et al., 2009; Singh et al., 2010; Cha et al., 2011; Doroshenko et al., 2014). The effects of antibiotics can also be affected by the particular microenvironment of biofilm, such as acidic pH and low level of oxygen encountered in the deep layers of the aggregates (Siala et al., 2014). The cells within biofilm are generally less metabolically active than planktonic cells and therefore significantly less sensitive to mechanism of action by many antimicrobials targeting synthesis of macromolecules or metabolic pathways such beta-lactams and quinolones (Xie et al., 2005; Mascio et al., 2007). In addition, a percentage of cells within biofilm may be persister cells, which are transiently antibiotic tolerant without harboring genetic changes seen in antimicrobial resistance. The presence of antibiotics can *per se* induce persistence (Dörr et al., 2010; Kwan et al., 2013) and/or enhance biofilm formation, particularly at sub-Minimal Inhibitory concentrations (MIC) (Wang et al., 2010b; Kaplan et al., 2012; Ng et al., 2014; Lázaro-Díez et al., 2016) and thus lead to treatment failure. The *in vitro* determination of MICs is restricted to planktonic cells growing exponentially under conditions that are optimal for action of the drug but unlikely to be met in biofilm populations. *In vitro* and *in vivo* experiments demonstrated that the MIC and the minimum bactericidal concentration (MBC) for biofilm bacterial cells are usually much higher (approximately 10–10,000 times) than their counterpart planktonic cells (Hengzhuang et al., 2012; Marquès et al., 2015). The effective antibiotic MBC *in vivo* for biofilm eradication are therefore impossible to reach by conventional antibiotic administrations due to the toxicities and the side effects of antibiotics and the limitation of renal and hepatic functions. Combination of antibiotics with different killing mechanisms leading to synergism remains nowadays the best solution for the treatment of biofilm infections. Rifampicin and fosfomycin-based combinations have shown *in vitro* enhanced activities against biofilm embedded *Staphylococcus aureus* isolates (Tang et al., 2012, 2013) but their *in vivo* efficiencies still remain to be determined (**Table 1**).

**Table 1:** Effects of different antibiotics family against *Staphylococcus* biofilms.

Antibiotic		Species	Assay	Effect on biofilm	Reference
Beta-lactams	Penicillins and most cephalosporins	*Staphylococcus aureus*	*in vitro*	Induction of biofilm formation at Sub-MICs	Ng et al., 2014


					Kaplan et al., 2012
					Lázaro-Díez et al., 2016
	Ceftaroline			Bactericidal anti-biofilm activity after prolonged exposure	Landini et al., 2015
Rifampicin		*S. aureus*	*in vitro*	Anti-biofilm activity, synergistic with fusidic acid and tigecycline	Tang et al., 2013
		*S. epidermidis*		High anti-biofilm activity alone or in combination with vancomycin or daptomicin	Olson et al., 2010
Vancomycin		*S. aureus*	*in vitro*	Promotion of biofilm formation through an autolysis-dependent mechanism	Hsu et al., 2011
		*S. epidermidis*		Induction of eDNA release at sub-MICs leading to increased biofilm formation	Doroshenko et al., 2014
Daptomycin		*S. aureus*	*in vitro*	Induction of viable but non-cultivable cells in biofilm at low concentrations	Pasquaroli et al., 2014
				Anti-biofilm effect in monotherapy	LaPlante and Woodmansee, 2009
			*in vivo*	Prevention of the emergence of rifampin resistance mutants	Cirioni et al., 2010
Fosfomycin		*S. aureus*	*in vitro*	Anti-biofilm activity synergistic with linezolid or minocycline or vancomycin	Tang et al., 2012

*eDNA, extracellular DNA; MIC, minimal inhibitory concentrations.*

In addition, biofilm phenotype provides resistance to host defenses, in particular, leukocyte phagocytosis. There are various possible mechanisms of action by which bacteria escape from the immune system including inhibition of inclusion of biofilm cells by phagocytes (Günther et al., 2009) and low immunogenicity of the biofilm matrix (Thurlow et al., 2011). These resistance properties and the genetic and phenotypic versatilities of cells within biofilm prompted workers to look for biofilm-specific therapies to eradicate this common cause of persistent infections. The term “anti-biofilm” appeared in the literature during the 1990s and is now widely used without, however, being fully defined. On the basis of current knowledge, this review proposes the use of the term anti-biofilm as “a natural or induced process, leading to reduction of bacterial biomass through the alteration of biofilm formation, integrity and/or quality”.

Studies have shown that two different anti-biofilm mechanisms are able to modulate biofilm formation: inhibition of bacterial surface attachment and destabilization/disruption of mature biofilms irreversibly attached. Many of the existing anti-biofilm agents are non-biocidal, but some bactericidal molecules could be considered as anti-biofilm agents as they are still active against mature biofilms protected by their architecture. Bactericidal anti-biofilm agents should be very specifically targeted otherwise their use could impair the composition of established ecosystems and damage beneficial microbiota. Nevertheless, anti-biofilm strategies represent interesting approaches for medical biotechnology as attested by the large number of recent publications (Rumbaugh and Ahmad, 2014). Anti-biofilm agents fall into two large groups, synthetic and natural (**Table 2**). Synthetic biofilm inhibitors, in part listed below, are mostly derived from bactericidal technologies.

**Table 2:** The different classes of anti-biofilm agents.

Synthetic	Natural product
Non-thermal plasma	Antibiotics
Photodynamic substances	Protozoan grazing
Nanoparticles	Plant products
Surface topographic modifications	Bacteriophages
Other peptides and molecules	Microbial agents

### Non-Thermal Plasma (NTP) Technology

Plasma is a unique state of matter that results from a rapid ionization of the gas obtained through subjecting gas to extremely high temperatures or passing gas through high-voltage electricity (Scholtz et al., 2015). The unspecific character of their anti-microbial activity, low toxicity for human tissues and absence of long-living toxic compounds make non-thermal plasmas (NTPs) a very promising tool for biofilm prevention and control in the decontamination of foods and biological materials (Ermolaeva et al., 2015).

### Photodynamic Substances

To produce an antimicrobial photodynamic therapy (PDT), three major components are needed, light, oxygen, and a photosensitizer. The excitation of the photosensitizer by light generates reactive oxygen species (ROS), which leads to the oxidation of biomolecules of microorganisms and results in cell damage and death (Hamblin and Hasan, 2004). It was recently shown that the photosensitizer 5-aminolevulinic acid (5-ALA), once absorbed by proliferating bacteria, is converted into the natural photosensitizer Protoporphyrin IX (PpIX), which has synergic effects with the antibiotic gentamicin against the biofilm of several Gram-positive bacteria (Barra et al., 2015). For example, photoactive TiO_2_ antibacterial coating was proposed to reduce pin tract infections and proved to have antibacterial effect against *Staphylococcus* strains (Villatte et al., 2015).

### Nanotechnology

Two kinds of nanotechnologies can affect biofilm formation on a surface. First, nano-modifications of surface topography (roughness and nanostructure) limit primary bacterial adhesion without the use of any biocide molecules (Desrousseaux et al., 2013). However, results on their efficacy have been conflicting and inhibition of primary adhesion seems to be dependent mostly on the spatial organization of the nano features. Second, surfaces can be chemically modified by addition of nanoparticles made of iron, silver, zinc, or titanium (Neethirajan et al., 2014). Most of these nanoparticles exert antibacterial activity by interacting electrostatically with the bacterial membrane, which leads to membrane disruption (Beyth et al., 2015). In addition, the bioavailability of these nanoparticles due to their high surface-to-volume ratios allows them to penetrate a mature biofilm and thus to target bacterial cells not only at the surface but also within the deep layers of biofilm (Bakkiyaraj and Pandian, 2014).

Other molecules could be added to this list, in particular detergents and antiseptics and synthetic peptides (de la Fuente-Núñez et al., 2014). However, the increasing interest in promoting health by natural means has concentrated research trends on natural biofilm inhibitor products with less biocidal activity.

### Protozoan Grazing

Protozoan grazing is believed to be the major trophic pathway whereby the biomass produced by bacteria, cyanobacteria and algae re-enters the food web. However, this type of microorganism’s biomass control is hard to adapt to human health. *In vitro*, the ciliate *Colpoda maupasi* has been shown to reduce the thickness of mature biofilms of opportunistic pathogens formed by *Klebsiella pneumoniae*, *Pseudomonas fluorescens*, and *Staphylococcus epidermidis* (Huws et al., 2005). In addition, the presence of protozoa in drinking water distribution systems can regulate the autochthonous and allochthonous bacterial populations (Sibille et al., 1998), which suggests that this process could be used to decrease or limit nosocomial infections caused by environmental contamination.

### Plant Products

Plants represent a huge resource of bioactive molecules. A recent study showed that some of them contain anti-biofilm compounds that inhibit growth, interrupt quorum sensing (QS) and/or prevent bacterial adhesion (Husain et al., 2015). Garlic acts as a QS-interfering compound in the treatment of bacterial infections, owing to the production of ajoene, a sulfur-rich molecule (Jakobsen et al., 2012). Cranberry is also an anti-adhesion agent (Allison et al., 2000; Cai et al., 2014) able to prevent urinary infections (Rafsanjany et al., 2015), dental caries (Girardot et al., 2014), and skin infections (Morán et al., 2014).

### Bacteriophages

The most abundant category of microorganisms on earth, are viruses whose interactions with biofilm members are ecologically important in horizontal gene transfer between bacteria (transduction). Bacteriophages play other important roles in microbial communities such as the modulation of bacterial populations. They also produce a number of enzymes able to disrupt the protection afforded by the biofilm matrix, thereby modifying biofilm architecture and increasing its susceptibility to antibiotics (Abedon, 2015). However, there are several drawbacks to the use of phages: (i) phage lytic activity releases Gram-negative bacterial-membrane-endotoxins, (ii) phage-resistant bacteria can arise rapidly (Örmälä and Jalasvuori, 2013) and (iii) phages can spread bacterial virulence genes (Rossmann et al., 2015).

Bioinspired anti-biofilm molecules can be isolated from eukaryotes, such as the lactoferrin (Ammons and Copié, 2013), but most are derived from microbial phenomena occurring within the biofilms themselves. In fact, bacterial fitness within biofilm relies on the ability of a given strain not only to adhere, settle, and develop as a biofilm, but also to inhibit others from doing so (Rendueles and Ghigo, 2015).

## Bacterial-Derived Biofilm Inhibitors

**FIGURE 2:**
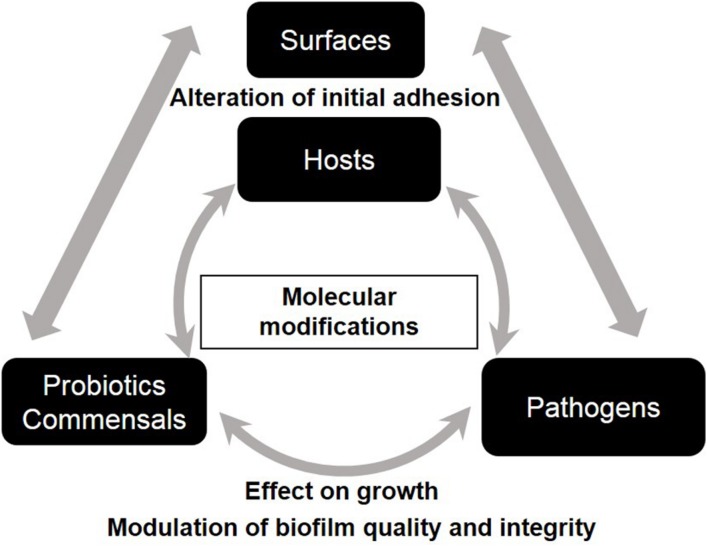
**Schematic representation of the impact of bacterial anti-biofilm activities on microbe-surface interactions and on microbe-microbe interactions**.

Intra- and interspecies interactions and competition between microorganisms within biofilm are governed by ecological and evolutionary parameters (Rendueles and Ghigo, 2015). Bacterial interferences are present at different levels of biofilm development; they can affect primary adhesion and/or maturation via exclusion/competition phenomena, modify matrix composition or enhance dispersal. Bacterial anti-biofilm activities govern microbe–surface interactions and microbe–microbe interactions and they are shared by commensal, pathogen, and probiotic bacteria (**Figure 2**). The increasing interest in promoting a natural approach to health has intensified research in the field of probiotics worldwide over the last two decades. Probiotics, recently redefined by an expert panel of the International Scientific Association for Probiotics and Prebiotics (ISAPP) as “live microorganisms that, when administered in adequate amounts, confer a health benefit on the host” (Hill et al., 2014), have gained increasing medical attention because of their antagonist effects against numerous pathogens. Probiotics with anti-biofilm properties, especially *Lactobacilli*, seem promising in the treatment of oral, wound and vaginal infections in both clinical trials and *in vitro* studies (Vuotto et al., 2014). For some probiotics, this beneficial activity is boosted when grown as biofilm (Rieu et al., 2014). Pathogens also exhibit anti-biofilm properties when competing with other bacteria to reach new ecological niches.

### Effect on Growth

Most beneficial and pathogen bacteria are able to secrete antibacterial substances such as antimicrobial peptides (AMPs), lantibiotics, bacteriocins, microcins, lactic acid, and hydrogen peroxide. Using different mechanisms of action, such as membrane permeabilization and interference with essential enzymes, these molecules inhibit bacterial growth or cause bacterial death. Some of them are highly effective against sessile cells such as the bacteriocins, nisin A, lacticin Q, sonorensin, and colicin R (Okuda et al., 2013; Rendueles et al., 2014; Chopra et al., 2015). Interestingly, colicin R produced by the commensal *Escherichia coli* ROAR029 strain, preferentially targets sessile rather than planktonic bacteria (Rendueles et al., 2014). The enhanced sensitivity of sessile cells to colicin R has been attributed to a reduced growth rate caused by diminished turnover of outer membrane components or increased stress within the biofilm. Several bacteriocin-producing strains have filed patent application for food and biomedical applications (Benmechernene et al., 2013). Interestingly, the probiotic lactobacilli strains *Lactobacillus casei* Shirota and *L. rhamnosus* HN001 inhibited growth and biofilm formation of the pathogen *Streptococcus mutans* by producing an acid environment and bacteriocin-like polypeptides, suggesting the synergic properties of these two mechanisms of action (Lin et al., 2015). Anti-biofilm agents impairing bacterial growth usually display narrow spectra. For instance, AMPs have shown a considerably stronger antimicrobial activity against biofilm formed by Gram-positive bacteria than by Gram-negative strains, justifying their potential use in the specific treatment of ocular infections, which are mostly due to Gram-positive bacteria (Dawgul et al., 2014).

### Alteration of Initial Adhesion by Surface Modification

The most effective strategy to antagonize the first step of biofilm formation is the use of biosurfactants and bioemulsifiers able to modify the physicochemical cell surface properties and thus to impair microbial adhesion. This is illustrated notably by the production of *Pseudomonas aeruginosa* rhamnolipids, which are able to disrupt the cohesiveness of biofilm formed by *Bordetella bronchiseptica* (Irie et al., 2005), *Bacillus subtilis*, *S. aureus*, and *Micrococcus luteus* (Quinn et al., 2013). Another anti-biofilm biosurfactant is the surfactin produced by *Bacillus subtilis*, which is able to reduce colonization of surfaces by the food pathogenic bacteria *Listeria monocytogenes*, *Enterobacter sakazakii*, and *Salmonella enteritidis* (Nitschke et al., 2009). Other pathogenic bacteria share such mechanisms: *K. pneumoniae* and the uropathogenic strain *E. coli* CFT073 exhibit a broad-spectrum anti-biofilm non-bactericidal activity by secreting extracellular polysaccharide (EPS) with anti-adhesion properties (Valle et al., 2006; Goncalves et al., 2014). EPS are the essential building blocks for the biofilm matrix of most microorganisms but they can also inhibit their neighbors’ biofilm structuration by interfering with initial adhesion, dispersion, cell-to-cell communication, and/or matrix formation (Rendueles and Ghigo, 2012). One example of this type of anti-biofilm mechanism of action is the interference of the capsular polysaccharides of *Actinobacillus pleuropneumoniae* serotype 5 with cell-to-cell and cell-to-surface interactions of other bacteria, which prevents them from forming or maintaining biofilms (Karwacki et al., 2013). More examples are given in the review of Rendueles and Ghigo (2012), in which the authors advise that targeting surface colonization rather than overall bacterial fitness is a more promising approach.

Homeostatic relations between the hosts and their microbiota considered as biofilms at the surface of epithelial host cells are abundant. At the intestinal level, commensal and probiotic bacteria strengthen intestinal barrier function by enhancing mucin production and tight junction integrity, and by modulating the activity of the immune system. These properties are assimilated to biotic surface modifications involved in the reduction of pathogenic-associated biofilm and thus the protection of the host from infections. For instance, the probiotic mixture VSL#3 is able *in vivo* to induce mucin gene expression (Caballero-Franco et al., 2007) and commensal bacteria such as bifidobacteria or *Bacteroides thetaiotaomicron* promote defense functions of the host epithelial cells via the production of acetate (Fukuda et al., 2012; Wrzosek et al., 2013). In contrast to these adaptations of host capacities, bacteria can specifically target the degradation of host receptors and then inhibit the adhesion process. For instance, some *S. epidermidis* strains secrete a serine protease named Esp, which degrades human receptor proteins (e.g., fibronectin, fibrinogen, and vitronectin) recognized by *S. aureus* and involved in host-pathogen interaction and tissue colonization (Sugimoto et al., 2013).

### Modulation of Biofilm Quality and Integrity

Evidence has shown that anti-biofilm bacterial agents not only modify biotic and abiotic surfaces but also alter the physical properties of bacterial surfaces involved in cell-to-cell aggregation and surface attachment processes. For instance, EPS released by *L. acidophilus* A4 exert anti-biofilm activity against a wide range of Gram-positive and Gram-negative bacteria by affecting the expression of genes involved in curli production and chemotaxis, and thus modifying cell-to-cell (autoaggregation) and cell-to-host cell (adhesion) adherence (Kim et al., 2009). More recently, inhibition of *S. aureus* was shown to be due to the physicochemical properties of the *Lactobacillus* cells surface such as hydrophobicity, autoaggregation, and coaggregation abilities (Ren et al., 2012). These kinds of inhibition processes are likely induced by alteration of the expression of key surface structures that are required for surface colonization and govern the complex interactions between pathogenic and/or common environmental bacteria. For instance, *Streptococcus intermedius* down-regulates the expression of both short (mfa1) and long (fimA) fimbriae required for attachment and biofilm development by *Porphyromonas gingivalis* (Christopher et al., 2010). The anti-biofilm activity of the oral strain of *Streptococcus* does not affect growth rate and is mediated by the surface arginine deiminase ArcA. More recently, it was shown that *Lactobacilli* strains impair fungal biofilm formation structure by down-regulating the expression of *Candida glabrata EPA6* and *YAK1* genes, encoding, respectively, an adhesin involved in the yeast biofilm development and its transcriptional regulator (Chew et al., 2015).

Anti-biofilm activities are characterized by inter-species communications not only between different genera of prokaryotes but also between prokaryotic and eukaryotic cells. Bacterial anti-biofilm activities are therefore likely to naturally regulate bacterial populations in an ecological niche. For example, the Esp protease secreted by a subset of commensal *S. epidermidis* in the nasal microbiota inhibit biofilm formation by pathogenic *S. aureus* (Iwase et al., 2010). Ecological homeostasis of polymicrobial biofilms involves exclusion, competition and displacement phenomena between pathogenic, commensal and/or probiotic bacteria for adhesion/attachment sites and/or nutriment access. A new mechanism of invasion resistance deployed by oral-derived microbial community (O-mix) to defend their domains was recently reported. The O-mix is able to restrict the colonization of exogenous *E. coli* strains by sensing the *E. coli* lipopolysaccharides (LPS) and subsequently killing them with oxygen free radicals (He et al., 2010). The underlying molecular mechanisms were recently discovered and involve the coordinated role of three commensal bacterial species (*Staphylococcus saprophyticus*, *Streptococcus infantis*, and *Streptococcus sanguinis)* acting as ‘Sensor,’ ‘Mediator,’ and ‘Killer,’ respectively (He et al., 2014). Numerous *in vitro* studies have shown that *Lactobacilli* can exert competitive exclusion of different pathogenic bacteria such as *S. aureus, Salmonella enterica, Shigella sonnei, E. coli*, and *Gardnerella vaginalis* by interfering with their binding sites on the epithelial cell surface (Jankowska et al., 2008; Prince et al., 2012; Zhang et al., 2012; Abedi et al., 2013; Castro et al., 2013).

Anti-biofilm activity can modulate biofilm bacterial diversity via interferences between species or between bacteria and the host surface. Different mechanisms of action can explain the chain reaction leading to the anti-biofilm process, the first of which is modification of cell-to-cell communication. The search for anti-biofilm compounds acting on QS and/or on signal molecule of targeted bacteria has already been undertaken (Leoni and Landini, 2014). For a recent review on QS inhibitors, see Brackman and Coenye (2015). It must, however, be noted that jeopardy of bacterial communication can lead to dispersion of a wide range of bacterial biofilms, and induction of biofilm dispersal by fatty acid signals may be a commonly used mechanism. For instance, the *cis*-2-decenoic acid produced by *P. aeruginosa* induces dispersion of *P. aeruginosa* PAO1 biofilm but also of those formed by a variety of Gram-negative and Gram-positive bacteria *(E. coli, K. pneumoniae, Proteus mirabilis, Streptococcus pyogenes, Bacillus subtilis, S. aureus*, and *Candida albicans*; Davies and Marques, 2009). Many bacterial enzymes involved in active biofilm dispersal have also been identified, in particular those involved in matrix degradation such as the serine protease Esp and the deoxyribonuclease I, DNase I. However, the most studied biofilm-matrix-degrading enzyme is dispersin B (Kaplan, 2009; Iwase et al., 2010; Brown et al., 2015). This glycoside hydrolase produced by the periodontopathogen *A. actinomycetemcomitans* completely inhibits biofilm formation and disperses preformed biofilm of several bacterial species: *E. coli, S. epidermidis, S. aureus, P. fluorescens, and Yersinia pestis* (Kaplan et al., 2004). As a consequence of the destruction of the physical integrity of the highly protective matrix barrier, sessile microbial cells are suddenly exposed to the external offensive of both antibiotics and innate host immune defenses (Kaplan, 2009). To avoid potential adverse effects due to the release of live bacteria from biofilms it seems essential to combine molecules with biofilm dispersing activity and anti-bacterial activity: *cis*-2-decenoic with antibiotics or disinfectants for eradication of catheter-associated biofilms (Rahmani-Badi et al., 2014; Sepehr et al., 2014) and dispersin B with KSL-W antimicrobial peptide for treatment of chronic wound infections (Gawande et al., 2014).

## Successful Use of Anti-Biofilm Methods in Human Health

Clinical trials performed with beneficial bacteria and particularly probiotics make use of exclusion and/or inhibition of growth of pathogens to protect the mucosa from the colonization of these undesirable microorganisms (Martín et al., 2014b). These strategies can be considered as anti-biofilm strategies, since aggregates formed on biotic surfaces, such as epithelia, have molecular properties similar to those of biofilms formed on abiotic surfaces and are actually considered as such (Stacy et al., 2016). However, most molecular knowledge of the biofilm mode of life derives from studies performed on aggregates formed on abiotic surfaces, and a lot remains to be discovered about the specific biofilm host-immune response. Most anti-biofilm based randomized clinical trials have focused on infections associated with biofilm formation on abiotic surfaces: medical devices (especially ventilator-associated pneumonia and catheter-related infections) or dental surfaces. In these randomized clinical trials, the main strategy used to successfully control biofilm formation was the use of surface-coated (Berra et al., 2008; Kollef et al., 2008) or surface-treated catheters (Scannapieco et al., 2009; Quintas et al., 2015) and/or changes in surface composition of the device (Ghorbanzadeh et al., 2015). The last paragraph of this review will focus on these different strategies, with special emphasis on their advantages and limitations.

## The Case of Catheter-Related Infections

Central venous catheters are essential in the management of patients and they are commonly used for the intravenous administration of fluids, blood products, complex drug treatments and total parenteral nutrition, for monitoring hemodynamics and for hemodialysis provision. The major concern with their use is colonization by microorganisms, which subsequently leads to infection, mostly catheter-related bloodstream infections (CRBSIs). CRBSIs are potentially devastating, entailing substantial morbidity, mortality and additional healthcare costs. It is estimated that a total of 250,000 cases of CRBSI occur yearly in the USA (Maki et al., 2006).

Catheter luminal colonization is the first step in catheter infection. In catheters with placement shorter than 21 days (short-term catheters), colonization originates mainly from the skin microbiota, with the microorganisms migrating distally along the external surface into the subcutaneous catheter tract. The microorganisms can also seed the intraluminal catheter surface from contaminated hubs, connectors and infusates. The intraluminal source of infection may be especially important in patients with long-term hemodialysis catheterization. Microbes colonizing catheter lumens constitute with host proteins a biofilm in which they proliferate and escape systemic antibiotics and immune host defenses. Ultimately, microorganisms can detach from biofilm and invade the bloodstream causing CRBSI and metastatic infections.

Multifaceted infection control interventions including maximal barrier precautions, line care bundle, development of educational programs, outcome surveillance and performance feedback of infection control practices have been implemented and succeeded in decreasing CRBSI rates. Several other preventive measures undertaken to further reduce the risk of CRBSI have been developed including coating catheter surfaces with antimicrobial agents and locking catheter lumens with antimicrobial solutions.

(1) **Coating catheter with anti-infective agents** (antiseptics or antibiotics) aims at inhibiting bacterial adhesion to the catheter lumen surface and preventing biofilm growth and subsequent infection. Catheters can be bonded to the inner and/or outer surface or impregnated within the material itself. The most commonly used antimicrobial agents are chlorhexidine-silver sulphadiazine (CHSS) and minocycline–rifampicin (MR).

First-generation antiseptic catheters whose outer surface was impregnated with CHSS had a lower rate of CRBSI when inserted for a short duration (<8 days) in units with a high incidence of CRBSI (>3 per 1,000 catheter-days; Walder et al., 2002). Second-generation CHSS catheters were then developed, with a long half-life of impregnation at the internal and external surfaces. Their use decreased the rate of catheter colonization but failed to diminish the incidence of CRBSI in units with acceptable rates of CRBSIs (Timsit et al., 2011). The development of bacterial resistance in relation to the use of CHSS catheters has never been observed in a clinical setting, but resistance to chlorhexidine has been detected in experimental studies (Tattawasart et al., 1999). Physicians must be aware that in rare cases hypersensitivity reactions have been reported in patients who were inserted with these catheters (Trautner and Darouiche, 2004).

Catheters impregnated intraluminally and extraluminally with MR have been widely developed. MR concentrations on the surface of these catheters decrease with placement duration but still exert antimicrobial activity through 60 days of catheterization (Darouiche et al., 2005). Their use is associated with a decrease in the rates of colonization and infection compared with standard catheters and first-generation CHSS impregnated catheters (Hockenhull et al., 2009; Wang et al., 2010a). No prospective trial has compared second-generation CHSS and MR-impregnated catheters. In a recent published retrospective study, second-generation CHSS and MR-impregnated catheters, in comparison with standard catheters, decreased the risk of CRBSI to a similar extent (Lorente et al., 2015).

There are concerns regarding the potential of MR-impregnated catheters for altering the microbiologic profile of catheter colonization/infection and promoting bacterial resistances. Data indicate that they can enhance the risk of fungal catheter colonization (León et al., 2004; Picioreanu et al., 2004; Lardon et al., 2011). However, bacterial resistance induced by the prolonged use of MR-impregnated catheters has not been demonstrated in clinical studies (Ramos et al., 2011; Bonne et al., 2015).

Other catheter materials have been tested including oligon, silver, carbon, platinum, and antimetabolite. Several prospective, randomized studies comparing these catheters with un-coated catheters in the prevention of catheter colonization and infection have yielded conflicting results (Kalfon et al., 2007; Walz et al., 2010; Lai et al., 2013).

Current evidence suggests the magnitude of the effect of antimicrobial-impregnated catheters differs according to the type of patient population. For instance, their beneficial benefit was mainly observed in critically ill patients and only inconsistently in cancer patients and in patients receiving total parenteral nutrition (Lai et al., 2013). The decision to use these catheters should be based on a risk benefit analysis taking into account the incidence of CRBSI observed in the institution for at-risk populations and balancing the attributable costs of CRBSI, the price over cost of impregnated catheters and concerns for the emergence of bacterial resistance.

Antimicrobial-impregnated catheters should be reserved for patients whose catheter placement is expected to be longer than 5 days, in units with unacceptable rates of infections (more than three CRBSIs per 1,000 catheter-days), despite adherence to a comprehensive preventive strategy (Bach et al., 1996).

(2) **The antimicrobial lock (AML) strategy** is designed to prevent or to treat endoluminal catheter infections. It is intended for catheters that are not used continuously and consists of instilling a selected AML solution into the catheter lumen while the catheter is idle. The AML solution is allowed to dwell or is “locked” for a certain period of time in the catheter lumen. Thereafter, the lock solution is aspirated and discarded or flushed through the catheter into the bloodstream. This strategy can achieve local antimicrobial concentrations 100–1,000 times higher than that obtained by parenteral treatment to overcome the adaptive bacterial resistance of sessile bacteria.

An ideal AML solution should possess several important properties including widespread bactericidal activity against the microorganisms commonly involved in CRBSIs, the potential to penetrate biofilm and kill sessile cells, prolonged chemical stability that does not impair catheter integrity, a low potential for promoting antimicrobial resistance, a low risk of toxicity and adverse events, and the ability to maintain catheter patency by preventing catheter occlusion and thrombosis. Thus, most AML solutions combine anticoagulant and anti-biofilm activities.

A wide variety of antibiotics have been used alone or in combination to lock catheter lumen, including penicillins, cephalosporins, aminoglycosides, fluoroquinolones, folate antagonists, glycopeptides, lipopeptides, oxazolidinones, rifampicin, polymyxins, tetracyclines, glycylcyclines, and carbapenems. The antibiotic solution is currently mixed in unfractionated heparin (UFH) to obtain antimicrobial-anticoagulant solutions. However, there is a growing body of data supporting the use of alternative anticoagulants such as low molecular weight heparins, calcium chelators (citrate or ethylenediamine-tetra-acetic acid [EDTA]), and tissue plasminogen activator. The choice of antibiotics and their concentration is based on the expected susceptibility of biofilm to the antimicrobials and their ability to kill the biofilm cells. In addition, the decision to choose a mixture must take into account the results of studies conducted on the stability and compatibility of the solution, which depend on the type of agents combined, their respective concentration and the experimental conditions including temperature, exposure duration, and storage conditions. Experimental studies suggest that antibiotic UFH mixtures are compatible with a broad range of antibiotic concentrations when unfractionated heparin concentration is higher than 3500 U/mL, while precipitation occurs when antibiotics are diluted with unfractionated heparin at concentrations lower than 1000 U/mL (Droste et al., 2003). Numerous data are now available to guide physicians in the choice of antibiotic lock solution components and their final concentrations (Mermel et al., 2009; Justo and Bookstaver, 2014).

The widespread use of the prophylactic antibiotic lock strategy raises concerns in clinical practice because of the risk for the development of antimicrobial-resistant organisms (Landry et al., 2010; Dixon et al., 2012). Emerging gentamicin resistant bacteria have been identified as causative agents of bacteremia in chronic hemodialysis patients when tunneled catheters were prophylactically locked with a solution of gentamicin (4 mg/mL) and unfractionated UFH (5000 U/mL; Landry et al., 2010). In addition, catheter locking may induce severe adverse events when a fraction of the lock solution spills from the catheter lumen into the bloodstream during and after instillation (Agharazii et al., 2005). Detectable gentamicin serum levels have been observed in chronic dialysis patients receiving preventive gentamicin-citrate lock (Dogra et al., 2002). Severe neurological disorders have been reported after prolonged exposure to aminoglycoside-based lock solution (Dogra et al., 2002; Saxena et al., 2002). The use of prophylactic antibiotic lock solution with antibiotics routinely used to treat systemic infections remains debatable, and in our opinion should therefore be discouraged.

One way to reduce the likelihood of antibiotic resistance is the use of catheter lock solutions that do not include an antibiotic component. The most commonly used non-antibiotic lock solutions are taurolidine, a high concentration of citrate or of EDTA and ethanol.

– Taurolidine is an amino acid taurine derivative with a broad-spectrum activity against bacteria and fungi. It acts as a disinfectant by inducing irreparable microbial cell wall injury (Willatts et al., 1995). Several randomized control studies have compared taurolidine and UFH lock solutions in preventing CRBSI (Betjes and van Agteren, 2004; Bisseling et al., 2010; Solomon et al., 2010; Dümichen et al., 2012; Handrup et al., 2013) and yielded mixed results depending on the study population. In pediatric oncology patients and infants on home parenteral nutrition, taurolidine used at various concentrations (1.35 and 2%) with or without addition of 4% citrate decreased the rate of CRBSI as compared to low doses of unfractionated heparin (150 and 100 U/mL; Bisseling et al., 2010; Dümichen et al., 2012; Handrup et al., 2013). In contrast, in hemodialysis patients, the use of 1.35% taurolidine – 4% citrate solution failed to prevent CRBSI and exit site infections as compared to UFH lock solution (5,000 U/mL), but increased the need for premature catheter removal for poor flow (Liu et al., 2014; Zhao et al., 2014).
– Citrate and EDTA have anticoagulant activities similar to those of heparin by chelating ionized calcium, which results in a blockade of the coagulation pathways. In addition, they enhance the activity of antimicrobial drugs and therefore there is a growing interest in the use of cationic chelator-based lock solutions in the prevention of CRBSI. These lock solutions have been widely studied and have been demonstrated to be highly effective in hemodialysis patients and pediatric cancer patients (Zacharioudakis et al., 2014; Zhao et al., 2014). In hemodialysis patients, the mixture of 7.0% sodium citrate, 0.15% methylene blue, 0.15% methylparaben, and 0.015% propylparaben reduced the risk of CRBSI in a randomized open label trial (Maki et al., 2011) but citrate alone was not more effective than UFH (Zhao et al., 2014). A similar result was observed with a lock solution of 4% EDTA (Kanaa et al., 2015). In addition, there have been concerns about the use of citrate at high concentrations owing to its potential toxicity, allergic reactions, arrhythmia and cardiac arrest (US Food and Drug Administration, 2000).
– Ethanol is an inexpensive antiseptic that acts by non-specific protein denaturation. It has drawn much interest as a lock solution for the prevention of CRBSI because it exerts bactericidal and fungicidal activity against a broad range of microorganisms and is unlikely to promote antimicrobial resistances. The time required by ethanol to eradicate experimental biofilm varies according to the microorganisms studied and is concentration-dependent. Ethanol has no antithrombotic properties and cannot be mixed with UFH because of potential precipitation. Randomized control studies on ethanol locks at concentrations higher than 70% v/v in the prevention of CRBSI have yielded conflicting results because of differences in study design, case mix population, and lock dwell time (Sanders et al., 2008; Slobbe et al., 2010; Broom et al., 2012; Pérez-Granda et al., 2014; Souweine et al., 2015). Either way, there are a number of concerns with the use of such high concentrations of ethanol in lock solutions for fear of catheter structural degradation, plasma protein precipitation and catheter occlusion (Mermel and Alang, 2014). However, ethanol solution at a 40% v/v concentration inhibits bacterial and fungi growth in established biofilms, does not induce catheter damage, and has satisfactory compatibility when mixed with low molecular weight heparin and heparinoids. When combined with low molecular weight heparin, 40% v/v ethanol may exert strong anticoagulant activity and has only a marginal impact on plasma protein precipitation. No clinical study has so far assessed the efficacy of such mixtures in preventing CRBSI.

## Future Challenges and Limitations of Anti-Biofilm Strategies

Conventional and current anti-biofilm therapies target one bacterial species without considering that most biofilm-related and chronic infections are due to the persistence of polymicrobial biofilms (Hall-Stoodley and Stoodley, 2009). Thus, there is no ideal solution to totally eradicate biofilm, but the key would be the simultaneous application of agents implementing mechanisms with synergic potential in order to both disturb the biofilm structure and kill bacteria (Khan et al., 2014). Multidisciplinary approaches are needed to decipher the generic networks underlying complex community interactions and to place them in their ecological and evolutionary context. The use of computational tools to comprehensively understand anti-biofilm processes seems essential. Biofilm and multispecies biofilm modeling techniques are available and take into account heterotroph parameters (Picioreanu et al., 2004; Lardon et al., 2011). Three-dimensional computer models of biofilm dynamics have been developed as tools for investigating mechanisms of protection against antimicrobial agents in biofilms (Chambless et al., 2006). They could be used for the analysis of the effects of anti-biofilm agents, in particular to assess their efficacy and to consider how they could impact the emergence of new classes of resistant microbes (Phillips et al., 2015).

The Food and Drug Administration (FDA) has received several medical device submissions that contain anti-biofilm claims (Phillips et al., 2015). However, current *in vitro* and *in vivo* assays are unable to effectively predict biofilm outcomes in humans and it is therefore important to develop reliable alternatives to clinical studies for the evaluation of anti-biofilm claims with standardized anti-biofilm procedures and validation methods that can establish correlation with clinical outcomes. That goes hand in hand with the elucidation of the mechanisms of action of the numerous anti-biofilm and bactericidal agents described so far. As already proposed in the Nutrition and Health claims domain, considering bacterial anti-biofilm agents will be useful in the future to establish a framework to help academic and industrial communities to explore their potential in accordance with health and nutrition policy (Miquel et al., 2015). In part because of the administrative complexity of these approaches, other potential applications must be envisaged such as vaccine strategy. The vaccine against the oral bacterium *Fusobacterium nucleatum* that preferentially targets FomA, an outer membrane protein involved in bacterial co-aggregation, can be considered as a pioneer anti-biofilm vaccine (Liu et al., 2010). New and specific vaccines are needed but it is necessary first to more fully investigate the interactions between biofilm and the host immune system, a domain as yet unexplored (Bryers, 2008).

Finally, modification of gene expression of pathogens within biofilm by probiotic counterparts could represent an interesting anti-biofilm approach with a dual-purpose: to limit bacterial colonization and inhibit the expression of virulence factors. For instance, some *Lactobacilli* down-regulate the expression of the virulence genes of *S. mutans, S. aureus*, and *Salmonella enterica* (Das et al., 2013; Nouaille et al., 2014; Wu et al., 2015). In the literature, the biofilm mode of life is generally opposed to the virulence capacities of bacteria (Claret et al., 2007), suggesting that biofilm ensures that bacteria stay within a specific niche and that its destabilization induces adverse effects. Further studies are required to assess the *in vivo* benefit of anti-biofilm approaches that can guarantee to have therapeutic or prophylactic benefits and to be very specific, highly effective and environmentally safe. However, due consideration should be given to the comparative risks and benefits for the patient, in particular the potential side effects on beneficial bacteria of the host microbiota and the emergence of antimicrobial resistances (Phillips et al., 2015).

## Conclusion

The objective of the review was to give a better definition of the anti-biofilm activities involved in microbial crosstalk. The prevalence of biofilm is not only a significant problem for human health, but also in food and the food industry, and in water and sewage treatment, and warrants expenditure for the development of effective anti-biofilm strategies. Coating catheter surfaces with antimicrobial agents and locking the catheter lumens with antimicrobial solutions are two different approaches that have produced encouraging results with regard to catheter-related infections. A major obstacle will be to translate the use of original anti-biofilm agents into commercial reality, in part because of the necessity to develop specific drug delivery applications. It still remains a real challenge for scientists in this innovative cross-cutting research domain.

## Author Contributions

All authors listed, have made substantial, direct and intellectual contribution to the work, and approved it for publication.

## Conflict of Interest Statement

The authors declare that the research was conducted in the absence of any commercial or financial relationships that could be construed as a potential conflict of interest.
